# Exploring Functional β-Cell Heterogeneity *In Vivo* Using PSA-NCAM as a Specific Marker

**DOI:** 10.1371/journal.pone.0005555

**Published:** 2009-05-18

**Authors:** Melis Karaca, Julien Castel, Cécile Tourrel-Cuzin, Manuel Brun, Anne Géant, Mathilde Dubois, Sandra Catesson, Marianne Rodriguez, Serge Luquet, Pierre Cattan, Brian Lockhart, Jochen Lang, Alain Ktorza, Christophe Magnan, Catherine Kargar

**Affiliations:** 1 Laboratoire de Physiopathologie de la Nutrition, Université Paris Diderot, CNRS UMR 7059, Paris, France; 2 Division Diabète et Maladies Métaboliques, Institut de Recherches Servier, Suresnes, France; 3 Institut Européen de Chimie et Biologie, Université de Bordeaux, CNRS UMR 5248, Pessac, France; 4 Division Pharmacologie et Physiopathologie Moléculaires, Institut de Recherches Servier, Suresnes, France; 5 Service de chirurgie générale, digestive et endocrinienne and Unité de Thérapie Cellulaire, Hôpital Saint-Louis, Paris, France; University of Bremen, Germany

## Abstract

**Background:**

The mass of pancreatic β-cells varies according to increases in insulin demand. It is hypothesized that functionally heterogeneous β-cell subpopulations take part in this process. Here we characterized two functionally distinct groups of β-cells and investigated their physiological relevance in increased insulin demand conditions in rats.

**Methods:**

Two rat β-cell populations were sorted by FACS according to their PSA-NCAM surface expression, *i.e.* β^high^ and β^low^-cells. Insulin release, Ca^2+^ movements, ATP and cAMP contents in response to various secretagogues were analyzed. Gene expression profiles and exocytosis machinery were also investigated. In a second part, β^high^ and β^low^-cell distribution and functionality were investigated in animal models with decreased or increased β-cell function: the Zucker Diabetic Fatty rat and the 48 h glucose-infused rat.

**Results:**

We show that β-cells are heterogeneous for PSA-NCAM in rat pancreas. Unlike β^low^-cells, β^high^-cells express functional β-cell markers and are highly responsive to various insulin secretagogues. Whereas β^low^-cells represent the main population in diabetic pancreas, an increase in β^high^-cells is associated with gain of function that follows sustained glucose overload.

**Conclusion:**

Our data show that a functional heterogeneity of β-cells, assessed by PSA-NCAM surface expression, exists *in vivo*. These findings pinpoint new target populations involved in endocrine pancreas plasticity and in β-cell defects in type 2 diabetes.

## Introduction

Pancreatic β-cells synthesize and release insulin with a remarkable degree of plasticity over time that allows adaptation to the metabolic environment. This involves increased β-cell function, *i.e.* an increase both in insulin production and secretion as well as enlargement of the β-cell pool [1]. The functional β-cell mass varies according to changes in insulin sensitivity. Thus, increased insulin sensitivity displayed by master athletes is associated with reduced insulin secretion [2]. On the contrary, insulin resistance as part of pregnancy or obesity is compensated via an increase in both β-cell number and responsiveness to glucose. This mechanism allows the maintenance of euglycemia in spite of decreased insulin sensitivity [3], [4].

The concept of functional heterogeneity among β-cells proposes that each cell differs in its sensitivity to glucose [5] and is recruited in a glucose-dependent manner into both biosynthetic [6], [7], [8] and secretory active states in order to adapt insulin secretion to the metabolic environment [5], [8], [9], [10], [11], [12]. Therefore characterization of such β-cell subpopulations with different metabolic sensitivities would lead to the development of new therapeutic strategies.

The sialylated form of the Neural Cell Adhesion Molecule (PSA-NCAM) is only expressed in structures undergoing functional changes in the adult such as brain and pancreatic β-cells [13], [14], [15]. In adult β-cells, the regulation of PSA-NCAM abundance at the cell surface is controlled by cellular activity, *i.e.* insulin exocytosis [15]. Interestingly, we have previously identified PSA-NCAM as a marker of β-cell functionality. According to the level of surface PSA-NCAM, β-cells were divided in two subpopulations with different glucose responsiveness in rats [13].

Our present study aimed to characterize these two groups of β-cells and to investigate the basis supporting their different insulin secretory capacities. Furthermore, we correlated the PSA-NCAM labeling to the functional β-cell mass in animal models where it is either decreased or increased: (i) the Zucker Diabetic Fatty (ZDF) rat, a model of type 2 diabetes [16] and (ii) the 48 h glucose-infused rat (HG/HI), a model in which a long-term imposed hyperglycemia leads to pancreatic overactivity and to an impressive increase of functional β-cell mass [17].

Taken together, our data (i) confirm that PSA-NCAM is a prominent marker of functional β-cells, (ii) provide proof of concept that a functional heterogeneity of β-cells exists *in vivo* and (iii) explore mechanistic insights of heterogeneity. Moreover, they support the idea that an alteration of pancreatic β-cell plasticity, *i.e.* an inability to recruit fully functional β-cells, could contribute to the impairment of insulin secretion in type 2 diabetes.

## Materials and Methods

### Animals

Experiments were carried out on 12 weeks old male Wistar rats and on 12 weeks old male Zucker Diabetic Fatty rats (ZDF fa/fa) and their age-matched lean littermates (ZDF fa/+). Animals were purchased from Charles River laboratories. Rats were housed under a 12 h light-dark cycle with free access to water. During the acclimatization period, animals were fed *ad libitum* on standard diet or on Purina 5008 chow (Charles River) for ZDF fa/fa rats. A subset of Wistar rats were infused with 30% glucose during 48 h to induce hyperglycemia and hyperinsulinemia (Wistar HG/HI) and compared to 48 h 0.9% NaCl-infused Wistar rats (Wistar control) as previously described [17]. *In vivo* insulin secretion in response to glucose represented by the insulinogenic index (ΔI/ΔG) was evaluated during oral glucose tolerance tests (OGTT). Insulin and glucose responses during OGTT (3 g/kg) were calculated as the incremental plasma insulin values integrated over a period of 30 min after the glucose gavage (ΔI) and the corresponding increase in glucose concentration (ΔG). The insulinogenic index represents the ratio of these two parameters. All procedures were performed according to the French ethical rules for animal experimentation.

### Islet cell preparation

Rats were anesthetized with pentobarbital (Sanofi; 4 mg/100 g body weight i.p.). Islets of Langerhans were isolated after collagenase digestion of the pancreas as previously described [18]. Islets were trypsinized with 0.1 mg/ml trypsin (1∶250, Difco) and digestion was stopped with cold Krebs-Ringer-Bicarbonate-Hepes (KRBH) buffer-0.5% BSA (Interchim) and 5.5 mM glucose [13]. Cell suspensions were used for immunocytochemistry or for β-cell sorting.

### Fluorescence-activated cell sorting of β-cells

Using a FACStar^plus^ (Becton Dickinson), β-cells were distinguished from non-β cells and sorted based on their autofluorescence (FAD content) and cell size, resulting in a population with 95% (insulin-positive) β-cells, as previously described [19], [20].

β-cells were analyzed for their surface PSA-NCAM expression using the mouse anti-PSA-NCAM antibody (AbCys) and the anti-mouse PE-conjugated secondary antibody (Invitrogren). The geometric mean fluorescence for PSA-NCAM was determined in control rats and used to arbitrarily separate between high PSA-NCAM-labeled β-cell population (β^high^-cells) and low PSA-NCAM-labeled β-cell population (β^low^-cells). In HG/HI and ZDF fa/fa rat models, the geometric mean of respective controls (ZDF fa/+ lean or NaCl-infused rats) was used to sort β^high^-cells and β^low^-cells.

Data were analyzed using the attached FACStar^plus^ analysis software (Becton Dickinson). Histograms are representative of acquisitions performed on 7500 events for Wistar control and HG/HI rats and 3500 for ZDF lean and fa/fa rats. Each experiment was performed on 1–2.10^6^ cells.

### Culture of sorted pancreatic β-cells

Sorted rat β-cells were resuspended in RPMI 1640 (MP Biomedicals) supplemented with 5.5 mM glucose, 10% heat-inactivated FCS, 100 µg/ml gentamycin, 2 mM L-glutamine and 10 mM Hepes and plated in miniculture dishes. For aggregates formation, β-cells were incubated during 1 h in a rotary incubator (30 cycles/min) at 37°C. Cells were then cultured for 18 h at 37°C in 95% O_2_/5% CO_2_ and saturated humidity and washed in KRBH buffer containing 5.5 mM glucose and 0.05% FAF-BSA (fraction V, Roche) before use. The cell viability rate was assessed by neutral red and was always between 90–95%.

### Insulin content

Freshly sorted β-cells were homogenized in acid alcohol solution (75% ethanol, 1.5% HCl 12N, 23.5% distilled water) and stored at −80°C until insulin determination.

### *In vitro* analysis of insulin secretion

*In vitro* insulin release was assayed either by perifusion or under static incubation on aggregated sorted β-cells (80×10^3^) after overnight culture.

The perifusion of rat cells was performed using low (5.5 mM) or stimulating (8.3 mM and/or 16.7 mM) glucose concentrations or KCl (50 mM) in KRBH buffer-0.05% FAF-BSA, as described previously [13]. The eluates were collected for insulin quantification. Incremental insulin response (ΔI) to stimulating glucose concentrations or KCl above basal release was obtained by planimetry of perifusion profiles and was expressed as the difference in insulin secretion rate relative to the mean hormonal output recorded at the pre-stimulation period with 5.5 mM glucose.

For static incubation, rat cells were pre-incubated for 60 min at 37°C in silicone-coated glass tubes in a shaking water bath containing KRBH buffer-0.05% FAF-BSA and 5.5 mM glucose. Thereafter, the medium was replaced by KRBH-BSA containing 5.5 mM or 16.7 mM without or with 10 nM GLP-1, 5 mM db-cAMP, 19 mM arginine or 10 mM leucine and β-cells were further incubated for 30 min at 37°C. The supernatant was stored at −20°C until assayed for insulin.

### Intracellular free calcium measurements

Overnight-cultured aggregated sorted rat β-cells (80×10^3^) were allowed to attach on polylysine-treated cover-glass then loaded for 1 h with 1.5 µM Fura-2/AM (Molecular Probes) at 37°C in Krebs-Ringer-Bicarbonate (KRB) buffer containing (115 mM NaCl, 5 mM KCl, 24 mM NaHCO_3_, 1 mM CaCl_2_, 1 mM MgCl_2_, 5.5 mM glucose and) 0.05% FAF-BSA. Thereafter they were transferred to a perifusion chamber placed on the stage of an inverted fluorescent microscope (Nikon Diaphot) and were perifused with 25 mM Hepes-buffered medium maintained at 37°C containing (125 mM NaCl, 5.9 mM KCl, 1.28 mM CaCl_2_, 1.2 mM MgCl_2_,) 0.1% FAF-BSA and supplemented with 5.5 mM or 16.7 mM glucose. The 340 to 380 fluorescence ratios (F340/F380) reflects the intracellular free calcium concentration [Ca^2+^]_i_ as previously described. The perifusion fluid was collected and stored at −20°C until assayed for insulin. Incremental Ca^2+^ response to stimulating glucose (ΔR) was obtained in the same manner as described above for insulin (ΔI).

### Insulin quantification

Insulin was measured using rat Ultra-Sensitive Insulin ELISA kits (Mercodia).

### Measurement of ATP content

ATP content was measured under static incubation simultaneously to insulin release on the same batches of β-cells (10^5^), in the β-cell pellets, using the ATP bioluminescence assay kit HSII (Roche).

### Measurement of cAMP content

cAMP content was measured under static incubation simultaneously to insulin release on the same batches of β-cells, in the β-cell pellets, by radioimmunoassay [21].

### Quantitative real-time RT-PCR analysis

Total RNA extractions were carried out immediately after sorting of rat β-cells with the RNeasy micro kit (Qiagen). RNA quality was assessed using the 2100 Bioanalyser (Agilent) on the basis of the RNA integrity number. Samples (1 µg) were then subjected to reverse transcription using the high capacity cDNA archive kit (Applied Biosystems). cDNAs (100 ng) were analyzed by real-time quantitative RT-PCR using TaqMan Universal PCR Master Mix (Applied Biosystems) on the 7900 HT Fast Real Time PCR system with a TaqMan low density array (Applied Biosystems). TaqMan gene expression assays (Applied Biosystems) were used as a set of primers and TaqMan probe for amplification of each gene of interest. Expression levels of target genes were normalized to 18s RNA. The assay ID for each gene is given in Table S1. Thermal cycle conditions were: 50°C 2 min, 94°C 10 min, followed by 40 cycles of 97°C 30 sec and 59.7° C 1 min.

### Western blot analysis

Rat β-cells were suspended in PBS-1% Triton X-100 containing a protease inhibitor cocktail (Sigma) and sonicated on ice. Proteins (20 µg/lane) were resolved on 4–20% linear gradient (for PSA-NCAM) and on 12% (for exocytosis proteins) SDS-PAGE gels and transferred to PVDF membranes (Amersham). Treatment of membranes, image acquisition and quantification was made as previously described [22], [23]. The following antibodies were employed: anti-PSA-NCAM (AbCys), anti-cyclophilin (Upstate), anti-syt9 (Beckton Dickinson), anti-Syntaxin1 (Sigma), anti SNAP-25 (Sternberger Monoclonals), anti-VAMP-2 (Synaptic Systems), HRP-linked anti-mouse (DAKO) and anti-rabbit antibodies (Amersham).

### Endoneuraminidase (EndoN) treatment of β-cells

Overnight-cultured aggregated sorted β^high^-cells (1.5×10^5^) were treated 1 h with 0.7 U endoN (AbCys) at 37°C to cleave PSA and subjected to western blot analysis for PSA-NCAM immediately after EndoN digestion or 3 h after EndoN digestion. EndoN treated β^high^-cells were tested for insulin secretion in response to 5.5 mM and 16.7 mM glucose in presence of EndoN to avoid PSA-NCAM reexpression, during static incubation studies.

### Immunolabeling on pancreas sections and isolated cells

Rat pancreata were fixed in 4% paraformaldehyde (PFA), immersed in PBS −30% sucrose and frozen in liquid nitrogen. For peroxydase labeling, successive tissue cryosections (7 µm thickness), fixed in acetone, were treated with anti-PSA-NCAM (AbCys) or anti-insulin (ICN) antibody overnight at 4°C. They were then incubated 1 h with biotin-conjugated anti-mouse (Vector) or with biotin-conjugated anti-Guinea pig (Jackson ImmunoResearch) antibody followed by incubation with HRP-labeled streptavidin (DAKO) and development using the Vectastain VIP or DAB kit (Vector).

Multiple immunofluorescence labeling for PSA-NCAM and islet hormones was performed on acetone-fixed rat pancreas cryosections. After overnight incubation with a mix of anti-PSA-NCAM (AbCys), anti-insulin (ICN), anti-glucagon (ICN), anti-somatostatin (ICN) and anti-pancreatic polypeptide (ICN) primary antibodies, sections were incubated 1 h with a mix of R-PE-conjugated anti-mouse (Invitrogen), FITC-conjugated anti-Guinea pig (Vector) or AMCA-conjugated anti-rabbit (Jackson ImmunoResearch) secondary antibodies. This multiple labeling was also performed on dissociated islet cells (50×10^3^) cultured on polylysine-coated cover-glass as previously described [13]. Images were analyzed with ImageJ (http://rsb.info.nih.gov/ij/).

### Confocal microscopy and quantification of actin filaments

Sorted rat β-cells (50×10^3^) were rinsed with PBS, fixed in 4% PFA and permeabilized in PBS, 0.2% triton X-100. Cells were washed, incubated with TRITC-phalloidin (Sigma) for 30 min, washed in PBS, mounted on slides and examined with a laser scanning confocal microscope (Leica SP2-AOBS). TRITC-phalloidin binds specifically to F-actin but not to actin monomers. Images of TRITC-phalloidin stained β-cells were collected under identical optical conditions and analyzed with ImageJ (http://rsb.info.nih.gov/ij/). A contiguous series of 28 optical sections (1 µm increments in the Z plane) was sufficient to capture most of the actin staining in the whole β-cell.

### Statistical methods

Data are reported as means±SEM. Statistical analyses were assessed by one-way ANOVA for comparison among multiple groups and by two-tailed Student's t test for comparison between two groups.

## Results

### Pancreatic β-cells are heterogeneous for PSA-NCAM

**Figure 1:**
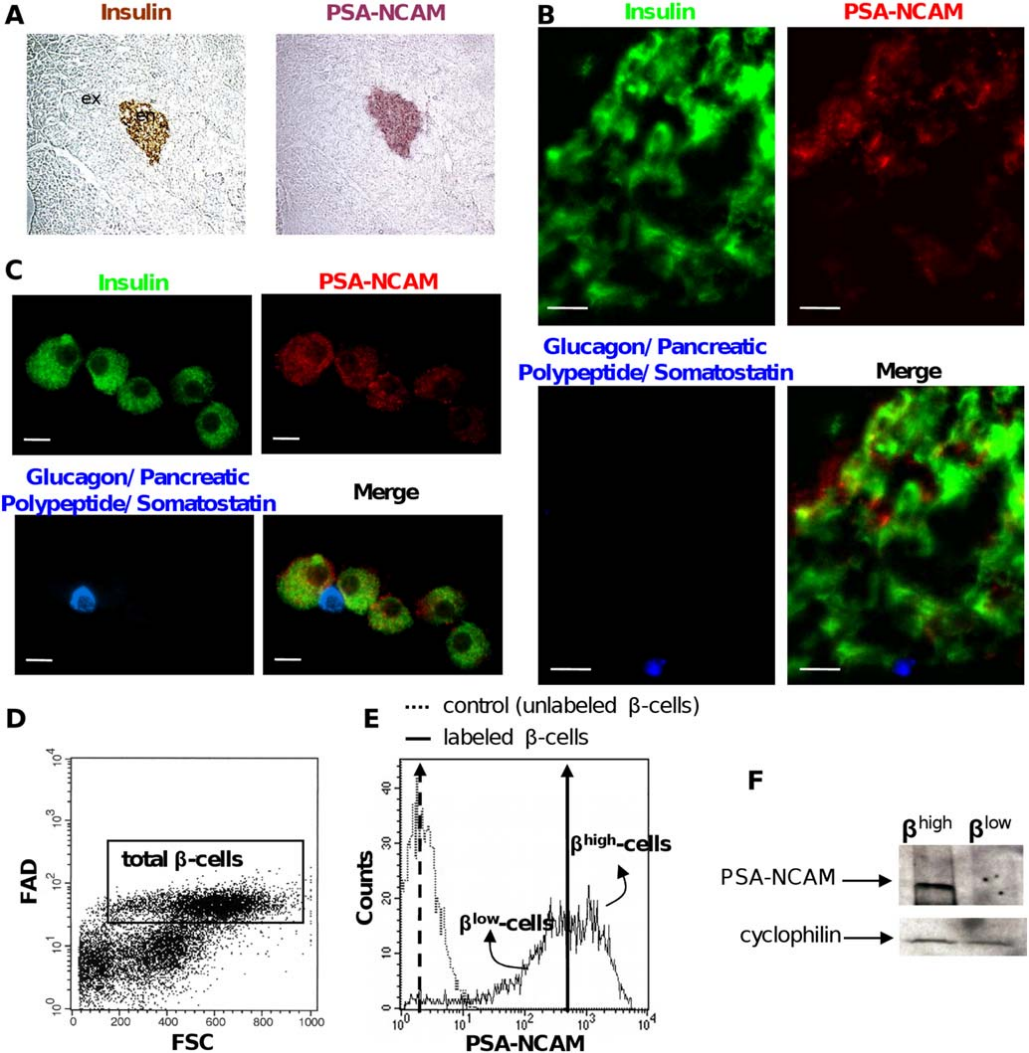
Pancreatic β-cells are heterogeneous for PSA-NCAM. (A) Immunochemical analyses on successive frozen rat pancreas sections stained for insulin (brown) and PSA-NCAM (purple). ex: exocrine tissue ; en: endocrine tissue. (B) Frozen rat pancreas. In islets, PSA-NCAM (red) colocalizes only with insulin (green) and not with other islet hormones (blue). Scale bar: 30 µm. (C) Dissociated islet cells. PSA-NCAM (red) colocalizes only with insulin (green) and not with other islet hormones (blue). Scale bar: 10 µm. (D) Representative dot plot analysis of dissociated islet cells examined for their FAD content and cell size (FSC) in order to gate total β-cells (black frame). (E) Representative histogram of PSA-NCAM surface labeling of total β-cells. Vertical arrows indicate the geometric mean (481±27 for labeled β-cells). This value was used to arbitrarily separate and sort highly (β^high^) and poorly (β^low^) PSA-NCAM labeled β-cells (n = 7). (F) Immunoblot analysis for total PSA-NCAM expression. Cyclophilin was used as loading control.

Staining of rat pancreas for PSA-NCAM and insulin showed that PSA-NCAM was exclusively located in islets and was not expressed in the exocrine tissue (Fig. 1*A*). Within islets, β-cells (in green, Fig. 1*B*) expressed different levels of PSA-NCAM (in red, Fig. 1*B*), i.e. they were heterogeneous for PSA-NCAM expression. PSA-NCAM was not observed in the other islet cells (in blue, Fig. 1*B*). Similarly, on dispersed islet cells, PSA-NCAM expression was specific of β-cells. In this case too, PSA-NCAM heterogeneity was obvious (Fig. 1*C*). This heterogeneity was further assessed by FACS analyses on isolated β-cells. Total β-cells were gated based on their FAD fluorescence and cell size (Fig. 1*D*), [19], [20] then analyzed for their PSA-NCAM surface expression. This latter was a broad range continuous spectrum, characteristic of heterogeneity (Fig. 1*E*). Using the geometric mean of fluorescence intensity (481±27 AU, black arrow in Fig. 1*E*), we arbitrarily divided the β-cell population into two groups of β-cells expressing high levels (β^high^-cells) and low levels (β^low^-cells) of surface PSA-NCAM (Fig. 1*E*). Sorted β^high^-cells exhibited higher levels of total PSA-NCAM (surface and intracellular) compared to β^low^-cells, as shown by immunoblotting (Fig. 1*F*).

### β^low^-cells are poorly responsive to glucose

**Figure 2:**
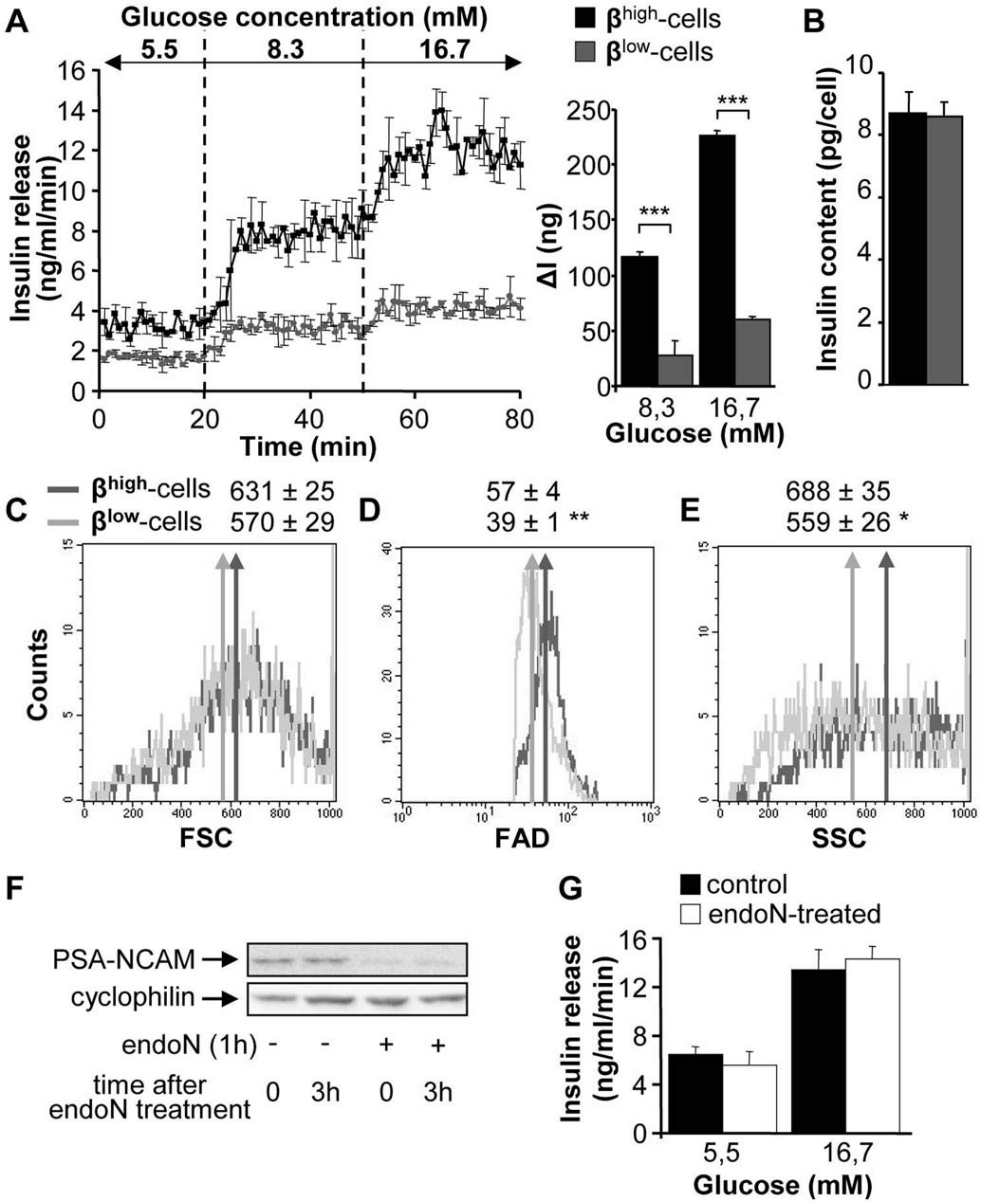
β^low^-cells are poorly responsive to glucose. (A) Insulin release in response to 5.5 mM, 8.3 mM and 16.7 mM glucose in β^high^ and β^low^ cells determined by perifusion experiments (n = 2). Quantification of insulin response to stimulating glucose is represented by ΔI. (B) Insulin content (n = 3). (C–E) Representative histogram of size (FSC) (C), FAD (autofluorescence) (D) and granularity (SSC) (E) of sorted β^high^ and β^low^-cells (n = 5–7). (F) Immunoblot analysis for PSA-NCAM expression after endoneuraminidase N (endoN) treatment (0.7 U/ml) of β^high^-cells. Cyclophilin was used as loading control. (G) Insulin release in response to 5.5 mM and 16.7 mM glucose in endoN-treated β^high^-cells (0.7 U/ml) assessed by static incubation experiments (n = 2). n represents the number of independent cell preparations from 6 pooled rats each. Data are means±SEM. *, p<0.05; **, p<0.01; ***, p<0.005.

We first investigated glucose-stimulated insulin secretion (GSIS) in β^high^ and β^low^ groups of cells. In β^high^-cells, increasing the glucose concentration from 5.5 mM to 8.3 mM and to 16.7 mM in the perifusion medium induced a marked increase in insulin release (Fig. 2*A*). In β^low^-cells insulin secretion was significantly lower than in β^high^-cells for all tested glucose concentrations (Fig. 2*A*). In the rest of the study, 5.5 mM and 16.7 mM glucose concentrations were used as low and high stimulating conditions. This difference in GSIS between β^high^ and β^low^-cells did not result from changes in insulin content (Fig. 2*B*) nor in cell size (Fig. 2*C*). However FAD and SSC parameters were significantly higher in β^high^-cells compared to β^low^-cells (Fig. 2*D–E*).

We next wondered whether PSA-NCAM itself was responsible for the difference of GSIS between the two groups of cells. To this end, sorted β^high^-cells were digested with endoneuraminase N (endoN) to remove PSA, as shown by immunoblotting (Fig. 2*F*). GSIS was similar in endoN-treated and non-treated β^high^-cells (Fig. 2*G*). Therefore the difference in GSIS between β^high^ and β^low^-cells can not be due to the sole difference of PSA-NCAM expression.

### β^low^-cells exhibit altered Ca^2+^ and ATP signaling in response to glucose

**Figure 3:**
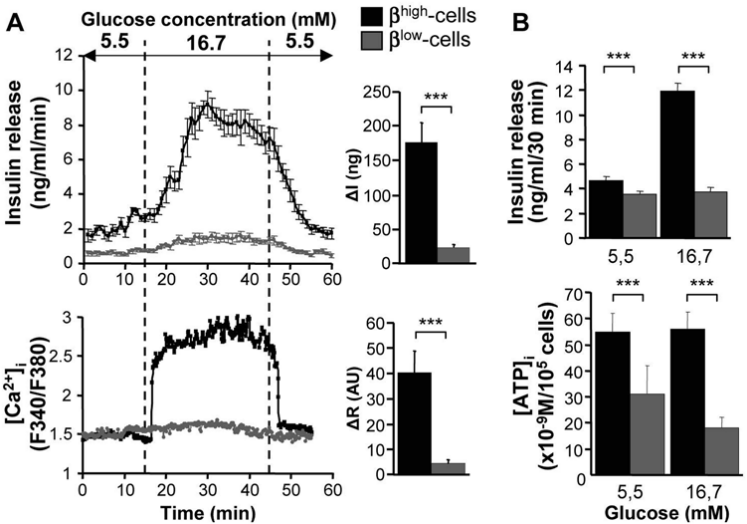
β^low^-cells exhibit altered Ca^2+^ and ATP signaling in response to glucose. (A) Insulin release (top panel) and cytoplasmic calcium concentration ([Ca^2+^]i) oscillations (bottom panel) in response to 5.5 mM and 16.7 mM glucose in β^high^ and β^low^-cells during perifusion experiments (n = 3–6). Quantification of insulin and calcium responses to stimulating glucose are represented by ΔI and ΔR respectively. (B) Insulin release (top panel) and intracellular ATP ([ATP]) levels (bottom panel) in response to 5.5 mM and 16.7 mM glucose in β^high^ and β^low^ cells assessed by static incubation experiments (n = 2). n represents the number of independent cell preparations from 6 pooled rats each. Data are means±SEM. ***, p<0.005.

In order to understand the differences between β^high^ and β^low^-cells, we investigated the intracellular messengers implicated in insulin secretion. Simultaneously to GSIS studies, the movements of intracellular calcium ([Ca^2+^]_i_) were evaluated. In β^high^-cells, 16.7 mM glucose triggered a typical [Ca^2+^]_i_ elevation that correlated, as expected, with the stimulation of insulin secretion whereas only a minor calcium response was observed in β^low^-cells (Fig. 3*A*).

Increased ATP levels being required for GSIS, ATP content were measured in both groups of cells. Increasing glucose concentration from 5.5 mM to 16.7 mM induced a GSIS in β^high^-cells but did not further increase the ATP levels, as previously described by others [24], (Fig. 3*B*). However ATP levels were higher in β^high^-cells compared to β^low^-cells at low and high glucose concentrations (Fig. 3*B*).

Thus, lower intracellular calcium movements and ATP levels contribute to impaired GSIS in β^low^-cells.

### β^low^-cells have an altered cAMP signaling

**Figure 4:**
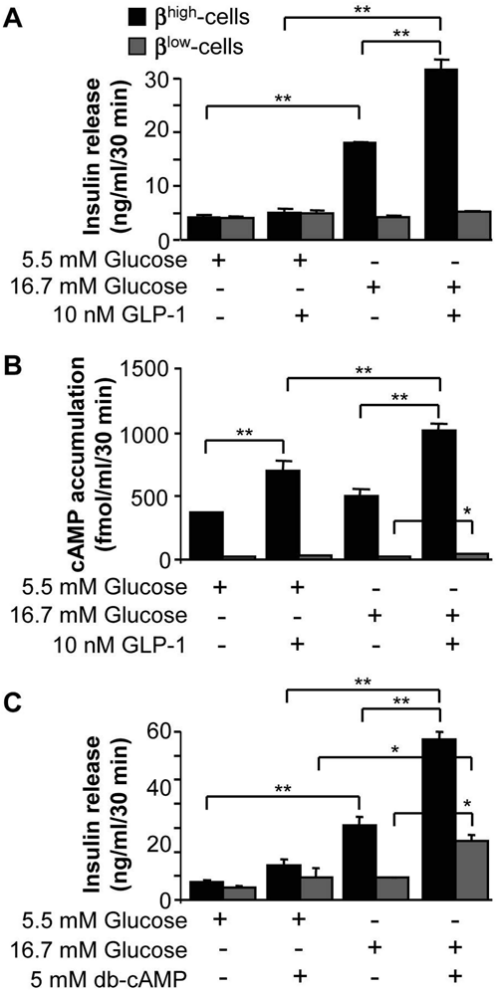
β^low^-cells have an altered cAMP signaling. Effects of 5.5 mM and 16.7 mM glucose±10 nM GLP-1 on insulin release (A) and cAMP content (B) in β^high^ and β^low^-cells assessed by static incubation experiments (n = 3–5). (C) Effects of 5.5 mM and 16.7 mM glucose±5 mM db-cAMP on insulin secretion in β^high^ and β^low^-cells (n = 3–5). n represents the number of independent cell preparations from 6 pooled rats each. Data are means±SEM. *, p<0.05 and **, p<0.01.

To investigate whether GSIS could be induced in β^low^-cells in the presence of a potentiator, we tested Glucagon Like Peptide-1 (GLP-1). At low glucose concentration, insulin secretion was similar in both groups and was not affected by the addition of GLP-1 (Fig. 4*A*). Insulin release in response to 16.7 mM glucose was potentiated by GLP-1 in β^high^-cells whereas the hormone was not efficient in β^low^-cells (Fig. 4*A*). As the GLP-1 receptor activates adenylate cyclase, we next investigated cAMP content as part of the signaling cascade. Indeed, cAMP levels were increased in the presence of GLP-1 at 5.5 and 16.7 mM glucose in β^high^-cells as compared to glucose alone. By contrast, cAMP content remained very low in response to glucose and/or GLP-1 in β^low^-cells (Fig. 4*B*).

Addition of dibutyryl-cAMP (db-cAMP), a cell permeable cAMP analog known to potentiate insulin secretion, significantly increased insulin release in β^high^-cells in the presence of 16.7 mM glucose (Fig. 4*C*). Although the addition of db-cAMP partially restored GSIS in β^low^-cells, insulin secretion was still lower than in β^high^-cells (Fig. 4*C*).

These observations indicate that β^low^-cells exhibit altered cAMP accumulation in response to GLP-1 signaling, consistent with the low ATP content.

### β^high^ and β^low^-cells respond equally to depolarizing agents but differently to metabolizable secretagogues

The difference in functional activity between both groups of cells may result from a metabolic or mechanistic failure or both. To investigate this point, β^high^ and β^low^-cells were treated with the metabolizable secretagogue L-leucine or the depolarizing agents L-arginine and KCl.

**Figure 5:**
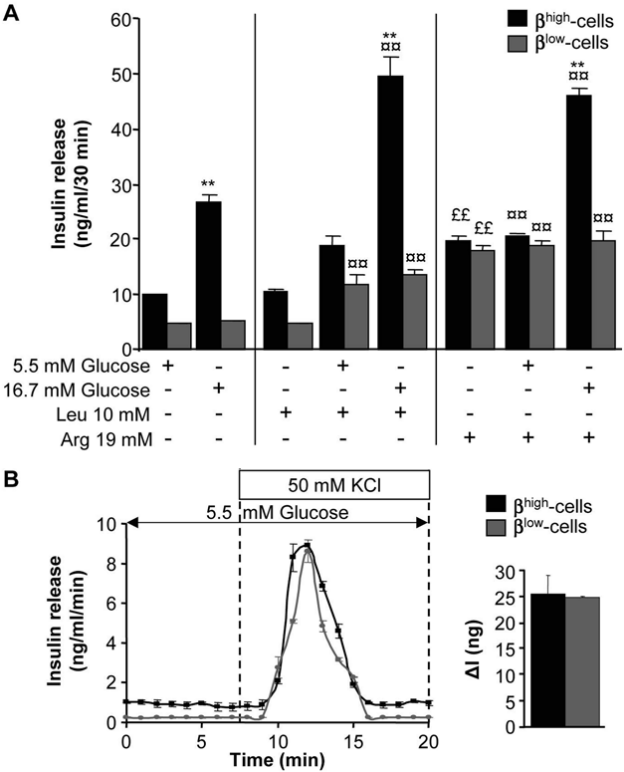
β^high^ and β^low^-cells respond equally to depolarizing agents but differently to metabolizable secretagogues. (A) Effects of 5.5 mM and 16.7 mM glucose±10 mM leucine (Leu) or 19 mM arginine (Arg) on insulin release in β^high^ and β^low^-cells (n = 3–5). Data are means±SEM. *, p<0.05 and **, p<0.01 for stimulating conditions (16.7 mM glucose±Arg or Leu) compared to basal conditions (5.5 mM glucose±Arg or Leu). ¤, p<0.05 and ¤¤, p<0.01 between treated (glucose+Arg or Leu) and non-treated conditions (glucose alone) for same glucose concentrations. ££, p<0.01 for Arg or Leu alone compared to 5.5 mM glucose alone. (B) Insulin secretory response to 5.5 mM glucose±50 mM KCl in β^high^ and β^low^-cells (n = 3–5). Quantification of insulin response to KCl is represented by ΔI. Data are means±SEM. *, p<0.05 between β^high^ and β^low^-cells. n represents the number of independent cell preparations from 6 pooled rats each.

Addition of 10 mM leucine alone elicited the same response as 5.5 mM glucose alone in both β-cell groups (Fig. 5*A*). When combined, the respective effects of 5.5 mM glucose and of leucine on insulin secretion were additive in β^low^-cells and in β^high^-cells. Whereas 16.7 mM glucose combined with leucine induced a stronger insulin release in β^high^-cells, it failed to further stimulate insulin secretion in β^low^-cells (Fig. 5*A*).

Unlike to leucine, 19 mM arginine stimulated insulin release in both β-cell populations and this response was significantly higher as compared to 5.5 mM glucose alone or 10 mM leucine alone (Fig. 5*A*). 5.5 mM glucose had no additive effect on arginine-stimulated insulin release. 16.7 mM glucose combined with arginine further stimulated insulin release in β^high^-cells but failed in β^low^-cells (Fig. 5*A*). Addition of 50 mM KCl led to an overall similar amount of insulin released in both groups (Fig. 5*B*).

These data suggest that metabolic alterations contribute to low insulin secretion in β^low^-cells.

### The exocytosis machinery of β^low^-cells is not favorable to GSIS

**Figure 6:**
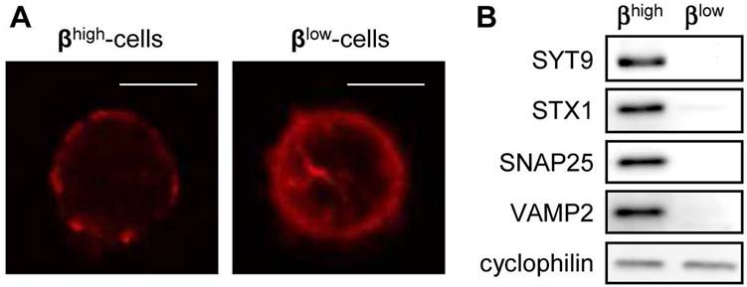
The exocytosis machinery of β^low^-cells is not favorable to GSIS. (A) Representative pictures of actin filament distribution in β^high^ and β^low^-cells. Scale bar: 10 µm (B) Representative immunoblots of exocytotic proteins in β^high^ and β^low^-cells (n = 3). n represents the number of independent cell preparations from at least 6 pooled rats each.

Exocytosis involves changes in the actin cytoskeleton and requires a number of conserved proteins. Confocal microscopy revealed a fragmented distribution of F-actin beneath the plasma membrane in 87±5% of β^high^-cells (Fig. 6*A*) whereas 78±7% of β^low^-cells displayed a strong and continuous signal potentially reflecting a ring beneath the plasma membrane that may hamper exocytosis (Fig. 6*A*) [25]. Proteins, such as SYT9, STX1, SNAP25 and VAMP2, required for exocytosis and its regulation by calcium were 3- to 5-fold less expressed in β^low^-cells as compared to β^high^-cells as shown by immunoblotting (Fig. 6*B*).

These data indicate that the exocytosis machinery of β^low^-cells is not favorable to GSIS.

### β^high^ *vs* β^low^-cells exhibit different gene expression profiles

We next investigated by RT-qPCR the expression of genes considered as highly representative of β-cell function, differentiation and survival (Table S1). The mRNA levels of transcription factors such as *Neurod1*, *Pdx1*, *Pax6* and *Nkx6.1* were 2.6 to 3.3 times more important in β^high^-cells than in β^low^-cells. Meanwhile, β^low^-cells expressed 2.6 to 3.6 higher levels of genes involved in early β-cell differentiation such as *Ngn3* and *Tcf7l2*. Regarding metabolism, some enzymes normally absent in β-cells, such as *Hk1*or *Ldha*, were highly expressed in β^low^-cells compared with β^high^-cells (12.1 to 16.7 times). At the same time several β-cell-related metabolic enzymes such as *Glut2*, *Gck*, *Pk*, *mtGPDH* and *Pcx* were expressed to a lesser extent in β^low^-cells (2.8 to 3.3 times). In addition, the levels of several pumps/ion channels (*Kir6.2*, *Sur1*, *Cav1.2*, *Kv2.1* and *SERCA2/3*) were 2 to 4 times higher in β^high^-cells compared with β^low^-cells. Similar results were obtained with genes implicated in the cAMP pathway (*Gcgr*, *Glp1r*, *PKA*, *Rap1a* and *Rab3a*). Expression of genes involved in insulin synthesis, maturation and secretion (*Ins* itself, *Iapp*, *PC1/3*, *PC2*, *Chga*, *Cx36* and *Slc30a8*) or exocytosis (*Munc13.1*, *Stx1a*, *Snap25*, *Vamp2* and *Rim2*) was downregulated in β^low^-cells up to 3.8 times compared with β^high^-cells.

This gene expression profile suggests that β^low^-cells are composed of immature and/or non-functional cells in contrast to fully functional β^high^-cells.

### The change in β^high^ to β^low^-cell ratio correlates with the change in β-cell function in animal models with increased insulin demand

**Figure 7:**
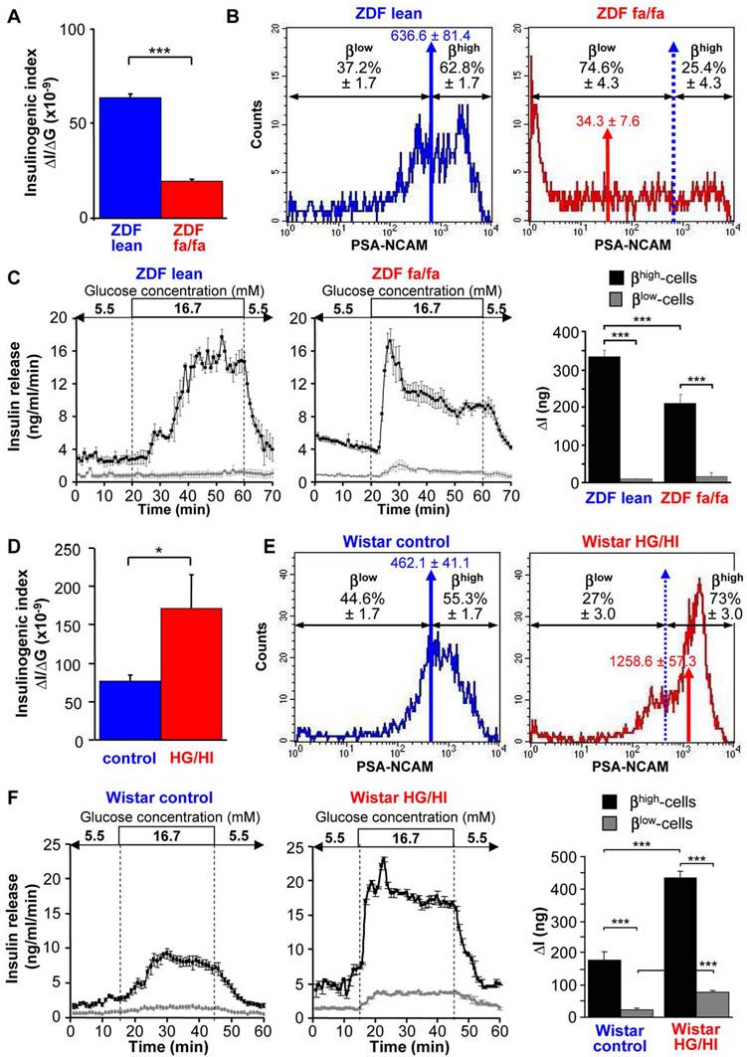
The change in β^high^ to β^low^-cell ratio correlates with the change in β-cell function in animal models with increased insulin demand. (A) Insulinogenic index (ΔI/ΔG) after OGTT in ZDF fa/fa rats compared to ZDF lean controls (12 rats). (B) Representative FACS analyses of dissociated islet cells in type 2 diabetic ZDF rat, a model of loss of β-cell function. The geometric mean of PSA-NCAM fluorescence is represented by a blue arrow in control ZDF lean rats and by a red arrow in diabetic ZDF fa/fa rats. The distribution of β^high^ and β^low^-cells is determined by the geometric mean of PSA-NCAM of the control ZDF lean rats (blue arrows) and expressed in percent of total β-cells (n = 3–6). (C) Insulin release in response to 5.5 mM and 16.7 mM glucose in β^high^ and β^low^-cells of ZDF rats during perifusion experiments (n = 3–6). Quantification of insulin response to stimulating glucose is represented by ΔI. (D) Insulinogenic index (ΔI/ΔG) after OGTT in HG/HI rats compared to saline-infused controls (Wistar control). (E) Representative FACS analyses of dissociated islet cells in HG/HI rats and Wistar control rats. The geometric mean of PSA-NCAM fluorescence is represented by a blue arrow in saline-infused Wistar control rats and by a red arrow in HG/HI rats. The distribution of β^high^ and β^low^-cells is determined by the geometric mean of PSA-NCAM of the control Wistar rats (blue arrows) and expressed in percent of total β-cells (n = 3–6). (F) Insulin release in response to 5.5 mM and 16.7 mM glucose in β^high^ and β^low^-cells of control and HG/HI rats during perifusion experiments (n = 3–6). Quantification of insulin response to stimulating glucose is represented by ΔI. n represents the number of independent cell preparations from at least 6 rats pooled each. Data are means±SEM. *, p<0.05; ***, p<0.005.

In order to evaluate the physiological relevance of PSA-NCAM distribution, we measured the proportion of β^high^ and β^low^-cells and their insulin secretory capacity in response to increased insulin demand in two animal models. We first used the ZDF fa/fa rat, a model for type 2 diabetes with impaired functional β-cell mass [16]. *In vivo* insulin secretion in response to glucose is markedly impaired in these animals at 12 week age, as reflected by a 70% decrease of the insulinogenic index compared with ZDF lean controls (Fig. 7*A*). The geometric mean of PSA-NCAM fluorescence of β-cells of ZDF fa/fa rats (red arrow, Fig. 7*B*) was strongly left-shifted compared to that of ZDF fa/+ lean controls (blue arrow, Fig. 7*B*), indicating a striking decrease of the expression of PSA-NCAM on β-cells of ZDF fa/fa rats (Fig. 7*B*). The β^low^-cell population was strongly predominant in ZDF fa/fa rats (74.6%±4.3% of total β-cells) in remarkable contrast to the age-matched ZDF lean controls in which the β^low^ population only represented 37.2%±1.7% of total β-cells (Fig. 7*B*). In addition, β^high^-cells from ZDF fa/fa rats were significantly less responsive to glucose compared to the ZDF lean controls (Fig. 7*C*) whereas the insulin secretion of β^low^-cells was unchanged (Fig. 7*C*).

The second model was the HG/HI rat in which the functional β-cell mass is improved [17]. The insulinogenic index was 2-fold increased compared with control saline-infused Wistar rats (Fig. 7*D*). The geometric mean of PSA-NCAM fluorescence of β-cells of HG/HI rats (red arrow, Fig. 7E) was strongly right-shifted compared to that of saline-infused Wistar rats (blue arrow, Fig. 7*E*), indicating a striking increase of the expression of PSA-NCAM on β-cells of HG/HI rats (Fig. 7*E*). β-cells were redistributed in favor of β^high^-cells, now representing 73%±3.0% of total β-cells in HG/HI rats as compared to β^high^-cells of controls (55.3%±1.7% of total β-cells) (Fig. 7*E*). β^high^ and β^low^-cells from HG/HI rats were more responsive than β^high^ and β^low^-cells of control rats (Fig. 7*F*).

These data show that the distribution of β^high^ and β^low^-cells correlates with physiological or pathological states regarding functional β-cell mass. Therefore surface expression of PSA-NCAM reflects the ability of the pancreas to secrete insulin.

## Discussion

The main outcome of our study is the demonstration of the existence of the functional β-cell heterogeneity *in vivo*. Based on the specificity of PSA-NCAM expression in pancreatic β-cells, we correlated this marker with β-cell functional activity in rats and explored the differences between both groups of cells.

β-cells are heterogeneous in their sensitivity to glucose and to non-glucidic nutrient secretagogues [5], [12]. Their metabolic redox state differs when stimulated with glucose and it is correlated with their capacity to secrete insulin [5], [8]. Recently expression of E-cadherin at the surface of β-cells was correlated with their insulin secretory capacity, promoting E-cadherin as a functional marker of β-cells [26]. However E-cadherin is expressed in both exocrine and endocrine tissues [26] in contrast to PSA-NCAM which is specific to β-cells as shown here. In the present study, using the arbitrary parameter of the geometric mean for PSA-NCAM fluorescence, we discriminated fully functional β^high^-cells from poorly glucose responsive β^low^-cells in rats, *i.e.* β^high^-cells were highly responsive to glucose whereas β^low^-cells showed weaknesses in insulin secretion.

The most striking difference between β^high^ and β^low^-cells is the small amplitude of glucose-induced raises in [Ca^2+^]_i_ and insulin secretion in β^low^-cells, reflecting a ticking over glucose metabolism in β^low^-cells. The effects of the metabolizable amino acid leucine were similar to those of glucose which reinforces the hypothesis of metabolic impairments in β^low^-cells. *Glut2*, *Gck*, *Pk*, *mtGPDH* and *Pcx* are metabolic enzymes implicated in the delivery of metabolites to mitochondria and thus the generation of metabolic signals, such as ATP, required for an appropriate insulin secretory response to glucose [27], [28]. The expression of these genes as well as the ATP levels were decreased in β^low^-cells while normally suppressed metabolic genes (*Ldha*, *Hk1*) were upregulated. Such alterations may be sufficient to interfere with glucose recognition mechanism by regulating metabolic pathways diversionary to normal β-cell metabolism [29]. β^low^-cells also showed deficiencies in cAMP-dependent pathways as evidenced by the use of GLP-1. Although the expression of the GLP-1 receptor and ATP content were diminished in β^low^-cells, the differential effects of db-cAMP also imply differences downstream of cAMP generation. Indeed expression of genes of the cAMP pathway such as *Gcgr*, *Glp1r*, *PKA* and *Rap1a* were decreased in β^low^-cells. Finally β^low^-cells expressed high levels of markers of progenitor cells *Ngn3* and *Tcf7l2* [30] while islet-associated transcription factors such as *Neurod1*, *Pdx1*, *Nkx6.1*, *Pax6* were downregulated. None of these transcription factors was completely shut off in β^low^-cells but modest reductions have wide repercussions [31], [32] including insulin secretion.

β^high^ and β^low^-cells also differ from a mechanistic point of view. First they showed different cellular complexity, implying differences in intracellular components. β^high^-cells exhibited a fragmented distribution of F-actin. Conversely β^low^-cells displayed a strong and continuous signal as a ring beneath the plasma membrane. This physical barrier modulates the access of insulin vesicles to the plasma membrane hence hampering exocytosis [25], [33]. In addition several proteins required in distal steps of exocytosis and its calcium regulation [34], such as SYT9, STX1, SNAP25 and VAMP2, were considerably downregulated in β^low^-cells as well as key pumps and ion channels such as *Kir6.2*, *Sur1* and *Cav1.2*, mediators of insulin secretion. The similar amount of released insulin in response to the depolarizing agents arginine and KCl may result from the fact that dynamics of insulin granules differs between K^+^ stimulation and glucose stimulation [35]. Indeed most of the granules responsible for fusion events induced by K^+^ stimulation consist of already docked granules to the plasma membrane whereas fusion in glucose stimulation implies newly recruited granules [35]. Collectively our data suggest that β^high^ and β^low^-cells may differ in their capacity to recruit new insulin granules to the cell membrane and that β^low^-cells are not able to properly respond to glucose in contrast to fully functional β^high^-cells. Therefore, in normal animals, PSA-NCAM is a specific marker to detect glucose responsive β-cells.

The physiological relevance of such functional heterogeneity is underscored by our findings in two animal models in which insulin demand is increased. In the HG/HI rat, a 48 h glucose infusion results in an increased functional β-cell mass [17]. In this model, insulin release in response to glucose of both β^high^ and β^low^ groups of cells was strongly enhanced compared to cells from saline-infused control rats. In the meantime the proportion of β^high^-cells rose to 75% of total β-cells compared to 55% in control rats. This expansion of fully functional β^high^-cells and of their capacity to release insulin easily explains the functional improvement in HG/HI rats [17]. It could be hypothesized that β^low^-cells may be a pool of cells recruited in presence of hyperglycemia as a component of endocrine pancreas plasticity. In ZDF fa/fa rats, a genetic model of type 2 diabetes with decreased functional β-cell mass [16], insulin release of β^high^-cells from ZDF fa/fa rats was strongly diminished compared to β^high^-cells from control ZDF lean rats. Moreover the distribution of β^high^ and β^low^-cells was completely inversed in ZDF fa/fa rats, poorly functional β^low^-cells becoming the predominant population (75% of total β-cells versus 37% in ZDF lean rats). These data perfectly fits with the failure of β-cell function in diabetic rats, *i.e.* impaired control of glucose homeostasis and onset of permanent hyperglycemia. Furthermore they suggest that an alteration of pancreatic β-cell plasticity, *i.e.* inability to recruit fully functional β-cells, is a key component in the development of type 2 diabetes [36]. Several reasons may account for the increased number of β^low^-cells in ZDF fa/fa rats. ZDF rats have a decreased β-cell mass due to massive apoptosis [37]. So the glucolipotoxic environment may induce the death of highly glucose responsive β^high^-cells. Otherwise, it may induce a loss of their activity favoring the loss of PSA-NCAM mobilization to the cell surface [15] which increases the number of β^low^-cells.

The follow-up of diabetic patients and anti-diabetic strategies requires reliable methods to assess the β-cell mass with non-invasive imagery procedures. Such approaches rely on the specific labeling of β-cells using enzymes, cell receptors or surface structures that are expressed on β-cells. Several β-cell structures, including GLP-1 receptors, sulfonylurea receptors, vesicular amin transporter 2 (VMAT2) or gangliosides have been used as labeling targets. However none of them has yet provided an accurate estimation of β-cell mass to justify a routine application in humans [38]. Because PSA-NCAM is a surface marker specific of functional β-cells at least in rat, it can be proposed as a potential tag to investigate functional β-cell mass by imagery approaches *in vivo*.

Here we characterized two groups of β-cells with different functional and gene expression profiles. Taken together the data suggest that β^low^-cells are poorly glucose responsive in contrast to highly responsive β^high^-cells. Moreover, the proportion of β^high^ and β^low^-cells varied according to the insulin demand and was correlated with the progression of diabetes. PSA-NCAM constitutes definitively a powerful functional β-cell marker which may provide further insight in β-cell plasticity and lead to the identification of new targets in order to improve the functional status of β-cells with low secretory capacities such as in advanced type 2 diabetes.

## Supporting Information

Table S1 β^high^ vs β^low^-cells exhibit different gene expression profiles. Gene expression profiles of β^high^ and β^low^-cells (n = 3). n represents the number of independent cell preparations from at least 12 pooled rats each. Date are means±SEM. *, p<0.05, **, p<0.01 and ***, p<0.005.
(0.04 MB DOC)
