# Supplementary material for: Exploring Functional β-Cell Heterogeneity In Vivo Using PSA-NCAM as a Specific Marker
Source: PLoS One. 2009 May 18;4(5):e5555. doi: 10.1371/journal.pone.0005555 (PMC2679208; doi:10.1371/journal.pone.0005555)
Supplement: Table S1 — βhigh vs βlow-cells exhibit different gene expression profiles. Gene expression profiles of βhigh and βlow-cells (n = 3). n represents the number of independent cell preparations from at least 12 pooled rats each. Date are means±SEM. *, p<0.05, **, p<0.01 and ***, p<0.005. (0.04 MB DOC) [file pone.0005555.s001.doc]

## Gene name Symbol Fold change p Assay ID

**(β^high^-cells *vs* β^low^-cells)**

# Transcription factors

Forkhead box 1 *Foxo1* + 2.4 ** Rn01494868_m1

Sterol regulatory element binding factor 1 *Srebp1c* + 1.7 * Rn01495766_m1

Siimilar to transcription factor 7-like 2, T-cell specific *Tcf7l2* - 3.6 * Rn01411019_m1

Eukaryotic translation initiation factor 2, subunit 1 alpha *Eif2s1* + 2.1 ** Rn00820979_g1

Nuclear Factor of Kappa light chain gene enhancer in B-cells *NFkB* + 1.7 ** Rn01399583_m1

Paired box 4 *Pax4* + 1.2 ns Rn00582529_m1

Paired box 6 *Pax6* + 2.7 ** Rn00443072_m1

Pancreatic and duodenal homeobox 1 *Pdx1* + 2.6 ** Rn00755591_m1

Neurogenin 3 *Ngn3* - 2.6 * Rn00572583_s1

NK6 transcription factor related, locus 1 *Nkx6.1* + 3.3 ** Rn00581973_m1

Carbohydrate response element binding protein *ChREBP* + 2.5 * Rn00591943_m1

cAMP responsive element binding protein 1 *Creb1* + 2.0 * Rn00578829_g1

Musculoaponeurotic fibrosarcoma oncogen A *Mafa* - 14.5 * Rn00824591_s1

Musculoaponeurotic fibrosarcoma oncogen B *Mafb* - 1.3 ns Rn00709456_s1

Neurogenic differentiation 1 *Neurod1* + 2.8 ** Rn00824571_s1

### *Receptors*

Insulin receptor *Insr* 0 ns Rn00567070_m1

Glucagon receptor *Gcgr* + 3.7 *** Rn00597158_m1

Glucagon Like Peptide-1 receptor *Glp1r* + 2.7 ** Rn00562406_m1

Somatostatin receptor 2 *Sstr2* - 3.5 *** Rn00571116_m1

Somatostatin receptor 5 *Sstr5* + 3.3 *** Rn00563577_m1

Insulin like growth factor receptor 1 *Igf1r* + 2.7 ** Rn00583837_m1

Endothelial growth factor receptor *Egfr* + 1.6 ns Rn00580398_m1

Brain derived neurotrophic factor receptor *Ntrk2* + 4.5 *** Rn00820626_m1

G protein-coupled receptor 40 *Gpr40* + 2.8 ** Rn00824686_s1

# Pumps / Ion channels

Solute Carrier Family 30 member 8 *Slc30a8* + 3.1 ** Rn00555793_m1

Solute Carrier Family 25 member 4 *Ant1* + 2.6 ** Rn00821477_g1

Potassium inwardly-rectifying channel, subfamily J, member 11 *Kir6.2* + 3.8 ** Rn01764077_s1

K+ voltage gated channel, Shab-related subfamily, member 1 *Kv2.1* + 2.3 * Rn00755102_m1

Inositol-3-P receptor type 3 *Itpr3* + 1.5 * Rn00565664_m1

ATPase, Ca++ transporting, cardiac muscle, slow twitch 2 *Serca2* + 3.0 ** Rn00568762_m1

ATPase, Ca++ transporting, cardiac muscle, slow twitch 3 *SERCA3* + 2.7 ** Rn00563800_m1

Ryanodin receptor type 2 *Ryr2* + 1.4 ns Rn01470303_m1

ATP-binding cassette, sub-family C (CFTR/MRP), member 8 *Sur1* + 2.6 * Rn00564778_m1

Caveolin 1.2 *Cav1.2* + 2.1 * Rn00709287_m1

# Hormones / Metabolism

Insulin *Ins* + 3.8 *** Rn02121433_g1

Glucagon *Gcg* - 2.4 ns Rn00562293_m1

Somatostatin *Sst* + 1.5 ns Rn00561967_m1

Amylin *Iapp* + 2.8 ** Rn00561411_m1

Chromogranin A *Chga* + 2.6 ** Rn00572200_m1

Glutamate dehydrogenase *Glud1* + 1.6 ** Rn00561306_m1

Lactate dehydrogenase *Ldha* - 12.1 *** Rn00820751_g1

Hypoxanthine-guanine phosphoribosyltransferase *Hprt* + 2.0 ** Rn01527840_m1

Carnitine Palmitoyltransferase 1a, liver *Cpt1a* + 1.6 ** Rn00580702_m1

Glycerol-3-Phosphate Dehydrogenase 2, mitochondrial *mtGPDH* + 2.8 *** Rn00562472_m1

Pyruvate carboxylase *Pcx* + 2.9 ** Rn00562534_m1

Pyruvate dehydrogenase kinase1 *Pdk1* + 1.1 ns Rn00587598_m1

Uncoupled protein 2 *Ucp2* + 2.5 ** Rn00571166_m1

Glucokinase *Gck* + 2.8 ** Rn00565467_m1

Phosphofructokinase 2 *Pfk2* + 3.0 * Rn00589696_m1

Hexokinase 1 *Hk1* - 16.7 *** Rn00562436_m1

Pyruvate kinase *Pk* + 3.3 * Rn00583975_m1

Glycogen synthase *Gys* + 1.2 ns Rn00565296_m1

Acetyl CoA carboxylase *Acc* + 1.6 ** Rn00573474_m1

Dipeptidyl peptidase 4 *Dpp4* - 7.6 0.06 Rn00562910_m1

Glucose transporter 2 *Glut2* + 3.3 ** Rn00563565_m1

Glucose transporter 1 *Glut1* + 2.3 *** Rn00593670_m1

Solute Carrier Family 25 member 12 *Aralar1* + 2.3 ** Rn01277989_m1

Dual specificity phosphatase 12 *Dusp12* + 1.8 * Rn00573828_m1

***Signaling***

Presynaptic cytomatrix protein *Pclo* + 2.6 ** Rn00571800_m1

FK506 binding protein 12-rapamycin associated protein 1 *mTOR* + 1.9 ** Rn00571541_m1

Protein kinase, cAMP dependent, catalytic, alpha Cs-*PKA* + 3.8 *** Rn01432300_g1

Proconvertase 1/3 *PC1/3* + 3.2 *** Rn00567266_m1

Proconvertase 2 *PC2* + 3.1 ** Rn00562543_m1

Thymoma viral proto-oncogen 2 *Akt2* + 1.6 * Rn00690901_m1

CREB regulated transcription coactivator 2 *Torc2* + 2.1 * Rn01455374_m1

Silent mating type information regulation 2, homolog 1 *Sirt1* + 1.5 ns Rn01428096_m1

Silent mating type information regulation 2, homolog 4 *Sirt4* + 1.9 * Rn01481485_m1

Activated mitogen protein kinase α2 subunit *AMPK* + 1.7 * Rn00576935_m1

Rad and Gem related GTP binding protein 2 *Rem2* + 2.9 *** Rn00575450_m1

Ribosomal protein S6 kinase, polypeptide 1 *Rps6kb1* + 1.5 * Rn00583148_m1

Rap guanine nucleotide exchange factor 4 *Epac2* - 3.1 *** Rn01514839_m1

Ras related protein 1A *Rap1a* + 2.0 * Rn01533681_g1

Calcium/calmodulin-dependent protein kinase 2δ *Camk2d* + 1.2 ns Rn00560913_m1

# Exocytosis / Adhesion

Gap junction protein delta 2 *Cx36* + 3.2 *** Rn00439121_m1

Integrin beta 1 *Itgb1* + 2.1 ** Rn00566727_m1

Regulating synaptic membrane exocytosis 2 *Rim2* + 2.3 ** Rn00588467_m1

Vesicle-associated membrane protein 2 *Vamp2* + 2.9 *** Rn00360268_g1

RAS-associated protein RAB3A *Rab3a* + 3.2 * Rn00564615_m1

Unc-13 homolog A *Munc13-1* + 2.5 ** Rn00575815_m1

Syntaxin 1a *Stx1a* + 1.6 * Rn00587278_m1

Synaptosomal-associated protein 25 *Snap25* + 3.0 ** Rn00578534_m1

Synaptotagmin like 4 *Sytl4* + 3.1 ** Rn00589676_m1

***Cell cycle / Apoptosis***

BCL2-antagonist of cell death *Bad* + 1.7 * Rn00575519_m1

Mitogen activated kinase 8 *JNK* + 2.4 * Rn01453358_m1

Casp8 and FADD-like apoptosis regulator *Flip* + 2.0 ** Rn00589205_m1

Tumor Necrosis factor Receptor superfamily, member 6 *Fas* - 2.7 * Rn00685720_m1

B-cell leukemia/lymphoma 2 *Bcl2* + 1.8 * Rn99999125_m1

Cyclin-dependent kinase inhibitor 2A *Cdkn2a* + 1.6 ns Rn00580664_m1

Cyclin-dependent kinase 4 *Cdk4* + 2.1 ** Rn00585909_m1

# Oxidative / RE Stress

DNA-damage inducible transcript 3 *Chop10* + 2.2 ** Rn00492098_g1

Gluthatione peroxidase 1 *Gpx1* - 1.5 * Rn00577994_g1

Heme oxygenase (decycling) 1 *Hmox1* - 2.4 * Rn00561387_m1

Superoxide dismutase 2 *Sod2* + 2.9 *** Rn00566942_g1

Eukaryotic translation initiation factor 2 alpha kinase 3 *Eif2ak3* - 1.1 ns Rn00581002_m1

Nitric oxide synthase 1 *Nos1* + 5.7 * Rn00583793_m1

# Miscellaneous

Cyclophilin A *Ppia* + 2.0 ** Rn00690933_m1

Cyclophilin B *Ppib* + 1.4 ns Rn00574762_m1

Thioredoxin 2 *Txn2* + 2.1 ** Rn00584162_g1

Thioredoxin interacting protein *Txnip* - 7.0 * Rn01533890_g1

Wolframine *Wfs1* + 3.2 ** Rn00582735_m1

WW domain binding protein 1 *Wbp1* 0 ns Rn01188847_g1
